# Different forms of informal coercion in psychiatry: a qualitative study

**DOI:** 10.1186/s13104-019-4823-x

**Published:** 2019-12-02

**Authors:** Veikko Pelto-Piri, Lars Kjellin, Ulrika Hylén, Emanuele Valenti, Stefan Priebe

**Affiliations:** 1 University Health Care Research Center (UFC), Faculty of Medicine and Health, Örebro University Hospital, Örebro University, House S, 701 85 Örebro, Sweden; 2 Population Health Sciences, Bristol Medical School, University of Bristol, Bristol, UK; 3 Unit for Social and Community Psychiatry, WHO Collaborating Centre for Mental Health Services Development, Queen Mary University of London, London, UK

**Keywords:** Psychiatry, Coercion, Informal coercion, Leverage, Ethics, Qualitative research

## Abstract

**Objectives:**

The objective of the study was to investigate how mental health professionals describe and reflect upon different forms of informal coercion.

**Results:**

In a deductive qualitative content analysis of focus group interviews, several examples of persuasion, interpersonal leverage, inducements, and threats were found. Persuasion was sometimes described as being more like a negotiation. Some participants worried about that the use of interpersonal leverage and inducements risked to pass into blackmail in some situations. In a following inductive analysis, three more categories of informal coercion was found: cheating, using a disciplinary style and referring to rules and routines. Participants also described situations of coercion from other stakeholders: relatives and other authorities than psychiatry. The results indicate that informal coercion includes forms that are not obviously arranged in a hierarchy, and that its use is complex with a variety of pathways between different forms before treatment is accepted by the patient or compulsion is imposed.

## Introduction

Coercion practiced under mental health legislation, often referred to as formal coercion, is subject to extensive international research [1–5]. There is also increasing research literature on other interventions, outside of formal coercion, in order to encourage reluctant psychiatric patients to accept treatment [6, 7]. In research focused on informal coercion, Szmukler and Appelbaum’s [8] hierarchy of treatment pressures—persuasion, interpersonal leverage, inducements, threats—is often referred to. Valenti el al [9] used case vignettes based on this hierarchy in a study of attitudes and experiences towards the use of informal coercion among mental health professionals in ten countries on the American continent and in Europe. They found that informal coercion, across different sociocultural contexts, is disapproved of in theory but nevertheless often used in practice.

Szmukler and Appelbaum’s [8] hierarchy consists of broad categories. Rugkåsa et al. [6] found three other categories of influencing behaviours practiced by community mental health professionals: building trusting relationships, negotiating agreements, and asserting authority. Considering that informal coercion seems to be widely used in psychiatry worldwide, and the ethical challenges this use implies, it is important to further enhance our understanding of informal coercion in clinical psychiatric practice.

The present study is based on focus group interview data from one of the participating countries in a previous international multi-centre study [9], namely Sweden. The objective was to further investigate how mental health professionals describe and reflect upon different forms of informal coercion. More specifically, the aims were to find out (1) how professionals argue around the treatment pressures presented by Szmukler and Appelbaum [8], and (2) if professionals identify, and if so how they reason about, other forms of informal coercion.

## Main text

### Methods

The methods of the international study are described elsewhere [9]. We used purposive sampling for gender, profession, and institution. In the Swedish part, three focus groups were recruited from general psychiatric services and one from a forensic psychiatric clinic, with staff from both in- and outpatient services located in three different cities. The four focus group interviews were carried out with five participants in each group. The participants were four mental health physicians, seven nurses and nine social workers, in all 13 women and seven men. Four participants had 1 to 5 years of working experience in psychiatry, eleven had 6 to 30 years and five had more than 30 years of experience.

A facilitator led the focus groups, assisted by a co-facilitator. An interview guide was followed and the discussion started with a general question about experiences of using coercive measures. Szmukler and Appelbaum’s [8] hierarchy of treatment pressures was presented and discussed. Thereafter the facilitator presented case vignettes structured according to this hierarchy, followed by a reflective discussion in the group about possible and acceptable forms of informal coercion (see Additional file 1).

Interviews were audiotaped and transcribed verbatim. We used qualitative content analysis [10, 11] to search for meaning units describing incidences of or reflections upon coercion of patients. Firstly, we searched with a deductive approach for the forms of pressure described by Szmukler and Appelbaum [8]. Secondly, descriptions and reflections about informal coercion that were not considered to belong to any of these forms were analysed inductively. We searched for all kinds of statements where staff expressed that they or someone else had restricted patient autonomy. All meaning units were coded and categorised. The original text was available during the whole process of analysis and we went back and forth between the whole and the parts of the descriptions whilst reducing the number of categories. In the final interpretation we created categories and subcategories considered close to describing the material as a whole.

### Results in the context of previous literature

In the deductive analysis we found several examples of persuasion, interpersonal leverage, inducements, and threats. This was expected since these forms of informal coercion were presented to the groups. *Persuasion* was described as commonly used, according to some participants in almost every consultation. Some participants did not accept that persuasion should be regarded as a form of informal coercion. They described that it sometimes could be more like a *negotiation*, reported by Rugkåsa et al. [6] as negotiating agreements.

Many participants described how *interpersonal leverage* could influence the decision-making process. Some of them said they used it almost all the time with patients with whom they had built up a confidence and an alliance, while others regarded it as wrong to use the personal relationship. Participants saw no problems in using small *inducements*, like an extra cigarette, coffee, or a walk, often as part of a negotiation in order to get the patient to accept medication, for instance. On the other hand, to use support or treatment as an inducement was regarded as very problematic. Some participants worried about that the use of interpersonal leverage and inducements risked to pass into *blackmail* in some situations.

In situations like the ones you brought up, suicide-threat situations, I think that on a number of occasions I’ve said: “I’d be really sad if you took your own life.” I’m using the therapeutic situation and there’s an element of blackmail, but there’s also an element of inducing guilt and shame. It’s not effective in the long run. I understand that. But in the short-term it can be very effective. You have to consider whether it’s OK to prevent the patient from doing this.

Participants described that when a patient after persuasion/negotiation did not accept a proposition they could immediately turn to *threats* of for example forced medication or involuntary admission. They discussed the intricate border between giving information and threatening the patient.

In the inductive analysis, we found three more categories of informal coercion. One was *cheating* the patient. Participants considered it wrong to give medicine without the patient being aware of it, but one participant reported having done that. When asked if this was acceptable, the answer was:

I want to say no, but I was the one that put the bloody tablet in the sandwich - so no. Then it’s all right then.

Strategic dishonesty and deception have been reported also in a study of psychiatrists’ experiences of consultations involving anti-psychotic medication [12], and Lidz et al. have previously identified deception as a form of coercion-related behaviour in the psychiatric admission process [13].

Another form of informal coercion found in our study was using *a disciplinary style*, like not saving any food if the patient was late for dinner or not allowing them to eat in the dining room when smelling bad. This form was not mainly used as a treatment pressure but rather as a pressure to adhere to societal norms and rules.

The third form was *referring to rules and routines*. Even voluntarily admitted patients may not be allowed to leave the ward without approval from the doctor, and the ward rules are the same for all patients.

My experience, in our department at any rate, is that there are quite a lot of things that we tell them. You can’t drink coffee whenever you want. You can’t smoke whenever you want. You can’t wear the clothes that you want to wear. This applies to voluntary and involuntary patients in our department. If anything, this is covert coercion. That those who shouldn’t even encounter coercion, do anyway to a great extent. I recognise this.

Participants also described situations of *coercion from other stakeholders*, namely relatives and other authorities than psychiatry. Relatives may threaten the patients to break the contact if they don’t accept the treatment offered.

The threats don’t just come from us; from the measures we use. Often they come from the patient’s nearest and dearest. “If you don’t admit yourself to hospital, if you don’t take your medication now, we’ll stop taking care of you.” Sometimes, these really are the toughest threats. They are already alone and they’re going to lose those who mean the most to them.

Regarding other authorities, participants described for instance that social services may demand that the patient undergoes a certain treatment in order to get financial support. This is in line with reports from the US and the UK of leverage from the social welfare and other systems [14, 15].

### Discussion and conclusions

Use of coercion implies ethical challenges and may cause moral distress and uncomfortable feelings among mental health care professionals [5, 9]. It is usually regarded as exercised on different levels on a continuum of coercion [16] or a hierarchy [8]. Our study indicates that in practice informal coercion may be used in a variety of ways, including but not exclusively limited to the treatment pressures in the commonly-used hierarchy, and not always starting with the least coercive step and if necessary moving on to the next. There seems to be a variety of more complex patterns of different combinations of pressures.

Apart from persuasion, interpersonal leverage, inducements and threats participants reported occasional use of cheating in order to get a patient to get medication. Trickery and cheating can be regarded as forms of influence strategies or covert coercion approaches. Shaw and Elger state the creation of new cognitive biases as an unacceptable form of persuasion, given the lack of transparency [17]. Interpersonal leverage and inducements could, according to some participants, turn into blackmail. Cheating and blackmail may be considered morally questionable and possibly contributing to moral distress among professionals. Other forms of informal coercion that we found, using a disciplinary style and referring to rules and routines, may be considered as belonging to the milder forms of coercion but may nevertheless impose infringements on patient autonomy. Informal coercion may also come from other stakeholders.

Another perspective is what the patients perceive as coercion. Many previous studies have shown that not only formally coerced patients but also patients in voluntary treatment may feel coerced (see for example [18]). This implies that different forms of informal coercion may or may not be perceived as coercion by patients.

Use of informal coercion may lead to the patient accepting treatment, so that the legal status of the patient is voluntary. If the patient does not accept treatment after applying one or more forms of informal coercion it will lead to the use of formal legal coercion (compulsion) in order to implement the treatment that is regarded as necessary. Informal coercion, mainly using a disciplinary style and referring to rules and routines, seems however not only to be used for treatment purpose but also to get the patient to behave in a socially acceptable manner. It has been found that for instance compulsory hygiene measures and different kinds of social activities may be perceived as coercion by patients [19].

Whether there is a hierarchy of pressures that is sequentially used by mental health professionals before formal legal coercion is applied has been suggested as an important research question [20]. Our results indicate that informal coercion includes forms that are not obviously arranged in a hierarchy, and that its use is more complex with a variety of pathways between different forms before treatment is accepted by the patient or compulsion is imposed. The use of informal coercion needs to be further explored in future research, but there is already an evident need for more debate, reflections and guidance regarding its use in mental health care [9].

## Limitations

The main limitation of the study is that our findings are based on data from a single country and a limited number of interviews.

## Supplementary information

**Additional file 1.** Focus group interview guide with two case vignettes

## Data Availability

The dataset generated and analysed during the current study are available from the corresponding author on reasonable request.
