# Supplementary material for: Different forms of informal coercion in psychiatry: a qualitative study
Source: BMC Res Notes. 2019 Dec 2;12:787. doi: 10.1186/s13104-019-4823-x (PMC6889621; doi:10.1186/s13104-019-4823-x)
Supplement: Supplementary file 1 — Additional file 1. Focus group interview guide with two case vignettes [file 13104_2019_4823_MOESM1_ESM.docx]

Additional file

***Focus group topic guide***

Sample introduction (5mins)

- Introduce self and any observer /other researcher
- Explain what focus group about, nature and purpose of research,  who research is for, briefly set out topics to be discussed
- Provide assurances about confidentiality
- Explain what happens to data collected – transcribing, reporting, anonymity
- Introduce and describe digital recording
- Set ground rules – one person to speak at time, everyone`s views important so want hear from everyone, no right or wrong answers, looking for range of views, consensus not required
- Mobile phone, blackberries etc. – off (or on silent/vibrate)
- Invite any question

Background (10mins)

*Aim: to provide demographic and other background details as a context for the main discussion*

- Introductory exercise (whether participants want to use their first names, last name or a pseudonym)
- Individually: ask each individual in turn to say a brief word about themselves
- Where they live
- Background
- Relationship to mental health services
- Collectively: ask whether they had some previous experiences with coercive measures in mental health such involuntary hospitalization, chemical restraint, mechanical restraint, isolation, persuasion, interpersonal leverage, inducement, threat.

General discussion (15mins)

*Aim: to explore professionals’ understanding of what the main topic is*

- Collectively: give an overview of the topic for discussion and ask for any initial thoughts
- Individually: ask whether they know the difference between formal and covert coercion

 Probe if not mentioned

- Persuasion
- Interpersonal leverage
- Inducement
- Threat
- Ask whether they know more kind of covert coercion than those mentioned
- Ask whether they think there exists a *continuum* between all kind of leverages

Present Vignette Cases Report (20mins)

*Aim: stress discussion on the description of cases and the most appropriate approach*

- Present case report 1and discuss about the type of coercion most appropriate
- What type of leverage is acceptable and appropriate?
- What aspects of the case need to be clarified or changed to make the different types of leverage acceptable and appropriate or not acceptable?
- What are the concerns if this patient is:
  - Dangerous for him-self or other
  - Neglected
  - There is any trust about clinical relationship cause the short-time
  - The patient has or has not a job or financial recourses
  - The patient has or has not a family support
- Finally further thoughts on the most appropriate approach towards the patient and conclusion (if there is enough data redundancy)
- Present Vignette Case Report 2 with the same structure for the discussion (if there is enough time to address a second case or any data saturation)

General Discussion (30mins)

*Aim: to explore professionals’ attitudes and views about leverages appropriateness and patient’s specificity*

- What is the impact of covert coercion on patient’s satisfaction?
  - How effective is this
  - What are the obstacles
- What is the impact of covert coercion on adherence to the treatment?
  - How effective is this
  - What are the obstacles
- There is a patient’s typology or clinical situation more appropriate for covert coercion?
  - Previously experience in involuntary hospitalization
  - Pathology
  - Acute symptoms
  - Autonomy
  - Other patient’s features
- There is the possibility to establish a link between patient’s typology and leverages typology?

Conclusion /Debrief (10mins)

*Aim: resume the discussion, stress opinions and perspective differences if not proceeds*

- Individually: ask each participant in turn if they agree or not with the resume perspectives and if there is anything they wish to add.
- Collectively: thank participants, ask them to complete brief feedback questionnaire including whether they would be happy to be contacted again to take part in another focus group
  - Ask if they have any further questions about the study.

***Vignettes for Focus Groups***

Case Report 1: continuum from persuasion to compulsion

*The patient is a 30-year-old woman with bipolar disorder who has had a number of admissions to hospital over the years, often as involuntary hospitalization. Between hospital treatments she keeps well and functions as long as she accepts medication and support. Without these she quickly becomes unwell.*

Persuasion

*The clinician in the out-patient service is increasingly concerned about the situation and keen to try and avert another damaging relapse. The clinician talks to the patient and explains the evidence for medication in bipolar disorder and the fact that her pattern of relapse indicates that this applies to her.*

Interpersonal leverage

*The clinician tries to appeal to the patient on the basis that they have known each other for a long time; he has always been there to help and would not advise her to do something that was not in her best interests.*

Inducement

*The appeals did not work and the patient is starting to show early signs of deterioration. There is a sale of children’s’ clothes coming up and the patient wants to buy something to give to her daughters when she next sees them. The clinician offers to give her a lift but says he can only do so if she is reasonably well. Whether or not the clinician means to imply she needs to take treatment in order to gain his assistance is left unclear, but that is the patient’s assumption.*

Threat

*The following week the patient is due to see her daughters. She is still refusing treatment and now shows signs of irritability, which for her is an early sign of relapse. The clinician explains that the access visit might have to be cancelled if she gets any more irritable or is still refusing treatment, and that he has a duty to let social services know about the situation.*

Compulsion

*The patient deteriorates further and becomes chaotic and angry. She is now at risk and is still refusing treatment and the clinician reluctantly arranges an involuntary hospitalization assessment. The patient is detained under the given legislation and taken to psychiatric hospital.*

Case Report 2: continuum from persuasion to compulsion

*The patient is a 40 year old man with chronic schizophrenia who lives in an independent flat with practically no any social contact and a tendency for self-neglect. He hears voices and believes neighbours are spying on him which makes him very distressed. In the past, he showed marked improvement when on medication. He has never harmed himself or others. He is willing to see staff of the community mental health, but not to take medication or leave the flat to participate in activities.*

Persuasion

*The clinician in the community team who has known the patient for a long time is concerned about the situation and keen to try and reduce the patient’s distress. The clinician talks to the patient and explains the importance of taking medication and engaging in social activities emphasizing that further refusal of treatment may lead to continuous or increased distress and impaired quality of life.*

Interpersonal leverage

*The clinician has repeatedly helped to prevent the patient from being evicted from his flat despite the obvious neglect and inconsistent payments of the rent. The clinician now says that it is frustrating to continue providing care and helping the patient unless the patient shows more engagement with treatment.*

Inducement

*The patient is keen on getting a new TV set, but can only afford it if social welfare provides the funding which requires an application that needs to be supported by the community team. The clinician brings this up and promises to help with such an application if the patient shows more engagement with treatment.*

Threat

*The patient has received another letter from the landlord with the intention to evict him from the flat. The clinician declares that the team will only help the patient to avoid eviction again if the he takes medication and/or regularly attends a drop in Centre for some structured activity and social contact.*

Compulsion

*The patient deteriorates further and is highly distressed all the time. The self-neglect has increased and he does not eat properly anymore. The team feels this is a serious threat to his health and arranges an involuntary hospital admission.*
